# Clinicobiological Characteristics and Outcomes of Patients with T-Cell Large Granular Lymphocytic Leukemia and Chronic Lymphoproliferative Disorder of Natural Killer Cells from a Single Institution

**DOI:** 10.3390/cancers13153900

**Published:** 2021-08-02

**Authors:** Andrea Rivero, Pablo Mozas, Laura Jiménez, Mónica López-Guerra, Dolors Colomer, Alex Bataller, Juan Correa, Alfredo Rivas-Delgado, Gabriela Bastidas, Tycho Baumann, Alejandra Martínez-Trillos, Julio Delgado, Eva Giné, Elías Campo, Armando López-Guillermo, Neus Villamor, Laura Magnano, Estella Matutes

**Affiliations:** 1 Department of Hematology, Hospital Clínic, 08036 Barcelona, Spain; anrivero@clinic.cat (A.R.); MOZAS@clinic.cat (P.M.); ABATALLER@clinic.cat (A.B.); JGONZALO@clinic.cat (J.C.); ARIVAS@clinic.cat (A.R.-D.); GNBASTIDAS@clinic.cat (G.B.); tsbauman@clinic.cat (T.B.); smartine1@clinic.cat (A.M.-T.); jdelgado@clinic.cat (J.D.); EGINE@clinic.cat (E.G.); ALOPEZG@clinic.cat (A.L.-G.); 2 Hematopathology Unit, Department of Pathology, Hospital Clínic, 08036 Barcelona, Spain; LJIMENE1@clinic.cat (L.J.); LOPEZ5@clinic.cat (M.L.-G.); dcolomer@clinic.cat (D.C.); ecampo@clinic.cat (E.C.); VILLAMOR@clinic.cat (N.V.); estela.matutes@sehh.es (E.M.); 3 Institut d’Investigacions Biomèdiques August Pi i Sunyer (IDIBAPS), 08036 Barcelona, Spain; 4 Centro de Investigación Biomédica en Red de Cáncer (CIBERONC), 28029 Madrid, Spain; 5 Department of Medicine, University of Barcelona, 08036 Barcelona, Spain

**Keywords:** T-cell large granular lymphocytic leukemia, chronic lymphoproliferative disorder of NK cells, outcome, *STAT3*

## Abstract

**Simple Summary:**

T-LGLL and CLPD-NK are two indolent lymphoproliferative syndromes whose main symptoms derive from the cytopenia(s). A proportion of patients harbor *STAT3* mutations that appear to play an important role in the pathogenesis of the disease. The aim of our retrospective study was to describe the main clinicobiological characteristics, response to therapy and outcome in a large cohort of T-LGLL and CLPD-NK patients diagnosed at a single institution. The impact of *STAT3* mutations on the behaviour of the disease and the survival of these patients compared to the age- and sex-matched general population are also evaluated. For the first time, we show that the survival of patients with these two diseases does not differ from that of a matched general Spanish population, confirming the indolent course of these entities.

**Abstract:**

T-cell large granular lymphocytic leukemia (T-LGLL) and chronic lymphoproliferative disorder of natural killer (NK) cells are two infrequent diseases characterized by clonal expansions of cytotoxic T lymphocytes and NK cells, respectively. Somatic mutations of *STAT3* are involved in the pathogenesis of these entities. We describe the clinicobiological features, mutational status of *STAT3/STAT5B*, treatment and outcome of 131 patients. Neutropenia was the most frequent finding at diagnosis, followed by anemia. Concurrent hematological disorders were diagnosed in 37% of patients and autoimmune conditions and solid tumors in 17% and 15%, respectively. All patients who needed treatment belonged to the CD8^+^CD57^+^ group. Remarkably, patients included in the CD4^+^ group had a higher association with solid tumors (*p* = 0.037). *STAT3* mutations were found in 17% of patients, mainly Y640F and D661Y mutations. Patients carrying *STAT3* mutations more frequently presented with anemia, neutropenia, high LDH, high large granular lymphocyte counts and need for treatment (*p* = 0.0037). Methotrexate was the most frequently used agent (72% of cases). The overall response rate to all treatments was 50%. The 10-year overall survival of this series was 78%, with no differences according to the mutational status of *STAT3*. We compared the survival of these patients with the general Spanish population and no differences were found, confirming the indolent nature of these hematological malignancies. Our study further extends findings documented by others on the clinical behavior of the disease and the impact of *STAT3*, and for the first time analyzes survival compared to a matched general Spanish population.

## 1. Introduction

Large granular lymphocytic leukemia (LGLL) is a rare lymphoproliferative disorder that results from the clonal expansion of lymphocytes with a characteristic morphological appearance, so-called large granular lymphocytes (LGL). The World Health Organization (WHO) classification recognizes two subtypes of LGLL based on the lineage of the leukemic cells: T-cell LGLL (T-LGLL) as a distinct definitive entity and chronic lymphoproliferative disorder of natural killer (NK) cells (CLPD-NK) as a provisional entity [1]. LGLL accounts for 2–5% of chronic lymphoproliferative disorders in North America and Europe and 5–6% in Asia, with around 85% of the cases corresponding to T-LGLL and 10–15% to CLPD-NK. Recently, two population-based studies have documented an incidence of 0.2 and 0.72 cases per one million individuals per year [2,3]. Both T-LGLL and CLPD-NK are indolent diseases. Initial manifestations are those derived from cytopenia, particularly recurrent infections related to chronic neutropenia. Less than half of the patients present with asymptomatic lymphocytosis, and a significant proportion of them have associated autoimmune diseases [4,5].

T-LGLL results from a clonal expansion of cytotoxic activated memory T cells resistant to activation-induced cell death due to constitutive survival signaling [1]. The pathogenic mechanisms that lead to resistance to cell death have not been completely elucidated. Information from molecular studies showing a disruption of the JAK-STAT signaling pathway has improved our understanding of the disease’s pathogenesis [6]. Activating *STAT3* mutations located primarily in exons 20 and 21 encoding the Src homology 2 (SH2) domain have been documented in 11–73% of T-LGLL and in 30% of CLPD-NK cases [6,7]. Less than 5% of patients harbor *STAT5B* mutations in the SH2 domain [8,9]. Whether *STAT3* or *STAT5B* mutations are correlated with specific clinicobiological features, their prognostic impact and response to immunomodulatory therapy have been poorly documented.

We describe here the main clinicobiological characteristics, response to therapy and outcome in a large cohort of T-LGLL and CLPD-NK patients diagnosed at a single institution. The impact of *STAT3* mutations on the behavior of the disease and the survival of these patients compared to the age- and sex-matched general population are also evaluated.

## 2. Materials and Methods

### 2.1. Patients

We included 131 patients diagnosed with T-LGLL or CLPD-NK between 1993 and 2018 at the Hospital Clinic of Barcelona (HCB). All the patients gave informed consent to participate in the study according to the declaration of Helsinki and the ethical standards of the Ethics Committee of the HCB. These samples belong to the Hematopathology collection (registered in the Biobanks of IDIBAPS-HCB (R121004-094) and correspond to the biological material remaining from the diagnostic samples at the Hematopathology Section, of HCB.

### 2.2. Diagnostic Criteria

The diagnosis of both conditions was established according to the revised WHO classification published in 2017 [1]. We retrospectively reevaluated all the patients to ensure that the diagnosis was correct according to the new criteria.

#### 2.2.1. Criteria for T-LGLL Leukemia

The diagnosis of T-LGLL was based on cell morphology and flow cytometry that demonstrated a cytotoxic T-cell phenotype. The clonal origin was investigated by a polymerase chain reaction (PCR) that demonstrated clonally rearranged TCR-γ and/or TCR-β genes, as previously described [10,11].

#### 2.2.2. Criteria for CLPD-NK

The CLPD-NK cases were identified through the characteristic NK cell phenotype (commonly surface CD3^−^, CD2^−^, CD8^+^, CD16^+^ and CD56^+^) in addition to the LGL morphology. Clonality was evaluated when available by expression of the killer-cell immunoglobulin-like receptor (KIR) family of NK-cell receptors. Either expression of a restricted KIR isoform or a complete lack of detectable KIRs was considered clonal.

Baseline patient characteristics were retrospectively assessed. Blood smear examination, flow cytometry analysis, bone marrow biopsy (when available) and molecular studies were reviewed in all cases to confirm the diagnosis. The antibodies used for the flow cytometry analysis are described in Table S1.

### 2.3. STAT3 and STAT5B Mutations

The hotspot regions of the SH2 of *STAT3* (codon 614 to 661) and *STAT5B* (codon 642 to 665) were analyzed by Sanger sequencing, as previously described [6,12].

### 2.4. Indications for Treatment, Assessment of Response and Survival

Indications for treatment included severe neutropenia (absolute neutrophil count < 0.5 × 10^9^/L), moderate neutropenia associated with recurrent infections, transfusion dependent anemia and associated autoimmune conditions requiring therapy [5]. Response to treatment was assessed 3–4 months after the beginning of treatment. Complete hematological response (CHR) was defined as the complete normalization of blood counts. Partial response (PR) was defined as an improvement of blood counts (hemoglobin >80 g/L, platelets > 50 × 10^9^/L and neutrophils > 0.5 × 10^9^/L) without complete recovery and the absence of transfusion requirements. Treatment failure was defined as a lack of any response or progression [4,13,14,15,16,17].

All patients were included for overall survival analysis, which was calculated from the date of diagnosis to the date of last follow-up or death from any cause.

### 2.5. Statistical Analysis

Differences between LGLL of T-cell origin and NK-cell origin and according to *STAT3* mutational status were assessed by using the Chi-square test (two-tailed), Fisher’s exact test, the Student’s *t*-test or nonparametric tests whenever necessary. Survival analysis was performed by the Kaplan–Meier method and differences assessed by the log-rank test. *p*-values < 0.05 were considered statistically significant. Finally, relative survival (RS) was analyzed with respect to a sex- and age-matched Spanish population (www.mortality.org accessed on 18 July 2020) using *R* software, version 3.3.2 (R Core Team, R Foundation for Statistical Computing, Vienna, Austria).

## 3. Results

### 3.1. Clinical Features

The median age at diagnosis was 67 years (range: 26–92 years), and 70 patients (53%) were women. In this case, 97 patients (74%) were diagnosed with T-LGLL and 34 (26%) with CLPD-NK. The clinical and biological characteristics of the patients are summarized in Table 1. Most of the patients were asymptomatic at the time of presentation. Splenomegaly was present in 12% of patients and a minority had hepatomegaly or lymphadenopathy. The median lymphocyte count and median absolute circulating LGL were 3.9 × 10^9^/L (range: 0.6–20.2) and 1.46 × 10^9^ (range: 0.12–18.98), respectively. Only 19 patients (14%) had an LGL count lower than 0.5 × 10^9^/L, of whom 14 (74%) had T-LGLL and 5 (26%) CLPD-NK. At diagnosis, about half of the patients had neutropenia and 25% anemia. Thrombocytopenia was the least frequent hematologic manifestation (17%). However, severe cytopenia (neutropenia < 0.5 × 10^9^/L, anemia < 80 g/L and thrombocytopenia < 50 × 10^9^/L) were only observed in a minority of the patients (Table 1). Only 12 patients (9%) required red blood cell transfusions, three of them due to a concomitant myelodysplastic syndrome. Two-thirds of patients had elevated β2 microglobulin levels and a small proportion had a raised LDH level. Recurrent infections were mostly related to neutropenia, but only 4% of patients had a severe infectious event leading to hospital admission. All these patients were diagnosed with T-LGLL. Most infections affected the respiratory tract, and one case was diagnosed with pseudomembranous colitis secondary to *Clostridium difficile* infection. No significant differences in clinical and biological features were observed between T-LGLL and CLPD-NK (Table 1).

Bone marrow biopsy was carried out for two reasons: patients in whom the diagnosis was unclear, and those requiring treatment. In this case, 28 patients had a bone marrow biopsy, and this could be evaluated in 24 patients (86%). Interstitial infiltration was the most common pattern found (67%).

### 3.2. Diseases Associated with T-LGLL and CLPD-NK

T-LGLL and CLPD-NK were associated with multiple conditions, as shown in Table 2. The commonest were hematological disorders, diagnosed in more than one-third of the patients. These included monoclonal gammopathy of undetermined significance (MGUS) (*n* = 20), lymphoproliferative disorders (*n* = 19) and acute myeloid leukemia/myelodysplastic disorders (*n* = 7). T-LGLL and CLPD-NK coexisted with solid tumors in 20 patients, with urologic tumors being the most frequent diagnosis. Autoimmune diseases were observed in 22 patients (21%), with rheumatoid arthritis (RA) and immune thrombocytopenia being the most frequent diseases, followed by autoimmune hemolytic anemia. RA was diagnosed prior to the onset of LGL leukemia in the majority of patients and all but one had a positive rheumatoid factor. Antinuclear antibodies (ANA) were positive in 28% of patients. Both T-LGLL and CLPD-NK followed an allogeneic stem cell transplantation and solid organ transplantation in 10% and 2%, respectively. One case of T-LGLL had primary pulmonary hypertension. As seen in Table 2, no differences in the associated condition between T-LGLL and CLPD-NK were found.

### 3.3. Characterization of Immunophenotype

The most frequent immunophenotype of T-LGLL was CD3^+^/TCR-αb^+^/CD4^–^/CD8^+^/CD57^+^, observed in 74% of patients. Uncommon phenotypes such as CD4^+^CD8^+^, CD4^+^CD8^−^ or CD4^−^CD8^−^/ TCR-γδ^+^ were identified in 18%, 5% and 3%, respectively. Cases with a CD4^+^CD8^+^ and CD4^+^CD8^−^ phenotype were considered as a CD4^+^ group for the purpose of this analysis. The main characteristics of the CD8^+^CD57^+^ and CD4^+^ groups are shown in Table 3. Only patients with CD8^+^/CD57^+^ required treatment (*p* = 0.032) and, remarkably, patients included in the CD4^+^ group more frequently co-occurred with solid tumors (*p* = 0.037).

The group of patients with CLPD-NK predominantly expressed a CD3^–^CD16^+^CD56^+^ phenotype (77% of cases). In 30 patients (88%), clonality was confirmed by restricted expression of KIR.

### 3.4. Mutational Status of STAT3 and STAT5B

*STAT3* mutation was analyzed in 119 patients. Mutations were detected in 20 cases (17%). The most frequent mutations were Y640F, present in nine patients (45%), and D661Y, in seven patients (35%). All the Y640F mutations were found in T-LGLL patients (seven cases with CD8^+^CD57^+^, one in CD4^+^ and 1 in CD8^−^CD4^−^), whereas D661Y was present in five patients with CD8^+^CD57^+^ T-LGLL and in two patients with CLPD-NK. The remaining four cases showed individual mutations: D661V, T620S and G618R in CLPD-NK cases and an 18 base pair insertion in the 657-codon exon 21 of *STAT3* gene in a T-LGLL case. The clinicobiological features according to mutational status of *STAT3* are shown in Table 4. Patients with *STAT3* mutations had a higher frequency of anemia (*p* = 0.046), neutropenia (*p* = 0.005), higher LGL counts (*p* = 0.039), raised LDH (*p* = 0.003) and therapy requirement (*p* = 0.042) compared to patients with wild-type *STAT3*.

The mutational status of *STAT5B* was determined in 42 patients, all of them wild type except one patient who showed N642H mutation.

### 3.5. Treatment

In this case, 18 patients received treatment (14%), including 13 of 97 patients with a diagnosis of T-LGLL (13%) and five of 34 with CLPD-NK (15%). Here, 13 patients (72%) received methotrexate (MTX), two (11%) cyclophosphamide (CP), two (11%) cyclosporine (CsA) and one CHOP as front-line therapy. MTX was given at doses of 10–15 mg/m^2^ p.o. weekly; CsA doses ranged from 50 to 100 mg p.o. bd and CP doses from 50 to 100 mg p.o. daily. All patients with hemolytic anemia received corticosteroid therapy. The overall response rate to all treatments was 50% (9/18) with a CHR in six patients and a PR in three patients. Primary refractoriness to first-line treatment was seen in seven patients (39%), while data were not available for two patients.

**Figure 1:**
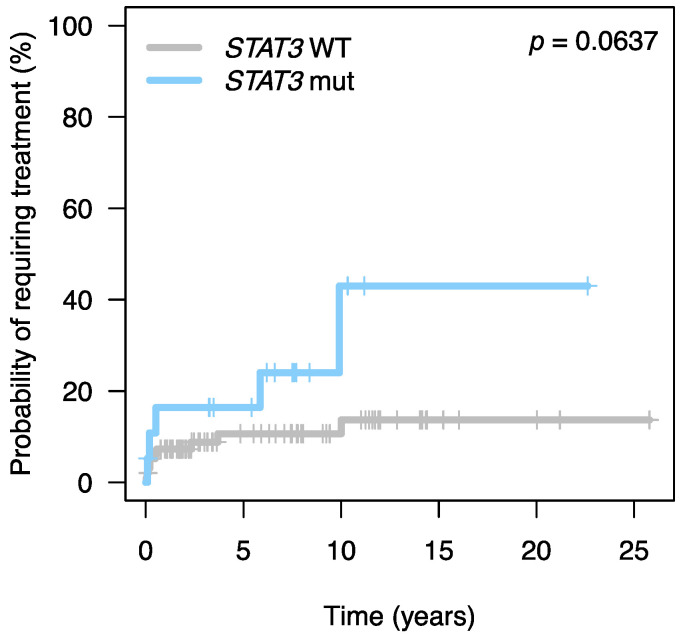
Time to first treatment according to mutational status of *STAT3*.

Overall, patients carrying *STAT3* mutations received treatment more frequently than those with the wild type (*p* = 0.042). In actuarial terms, the time to first treatment was shorter in patients with mutated *STAT3* than in those with the wild-type. (Figure 1).

Splenectomy was performed in 13 patients (10%). In four patients, it was carried out as a therapeutic approach, not frontline therapy; in three patients it was for therapeutic/diagnostic purposes of a concomitant B-lymphoproliferative disorder, in two patients it was performed for the management of immune thrombocytopenia, and in one patient splenectomy was required due to splenic rupture. Information was not available on the remaining three cases.

### 3.6. Survival

**Figure 2:**
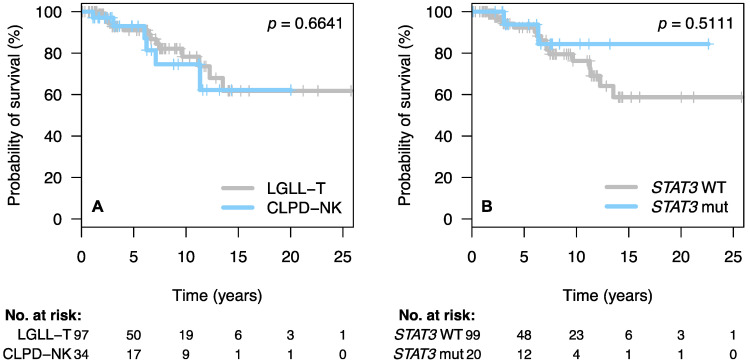
Overall survival of the patients of the series according to WHO diagnosis (**A**) and mutational status of *STAT3* (**B**).

After a median follow-up of 5.4 years (range: 0.11–25.78 years), 20 patients (15%) had died. The median OS for the entire cohort was not reached and the 10-year OS rate was 78% (95% CI: 72–83). In this case, 45 percent of deaths were due to neutropenia-related infections and 20% due to concomitant solid or hematological malignancies. The remaining 35% of deaths were unrelated to the LGLL, or information was not available. No significant differences were observed when OS was analyzed according to the phenotype (T-LGLL vs. CLPD-NK) (Figure 2A) or *STAT3* mutational status (Figure 2B).

**Figure 3:**
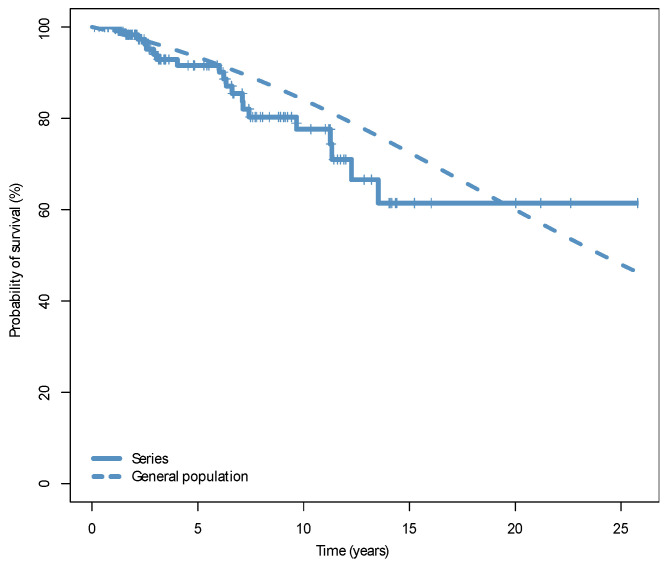
Overall survival of the patients of the series (continuous line) and survival of an age- and sex-matched Spanish general population (dashed line).

Finally, we compared the relative survival (RS) of T-LGLL and CLPD-NK patients with that of the sex- and age-matched general Spanish population. The 10-year RS was 93%, with no significant decrease in life expectancy with the current follow-up (Figure 3).

## 4. Discussion

We described our real-life experience (clinical features, response to therapy, impact of *STAT3* mutations and outcome) of 131 unselected patients with T-LGLL and CLPD-NK diagnosed and followed up at a single institution. To our knowledge, this is the first study to compare the survival rate of T-LGLL and CLPD-NK patients with that of a sex- and age-matched general Spanish population (relative survival). No differences were found, confirming the indolent nature of these hematological malignancies. The 10-year OS rate in our cohort of patients was 78% without differences when we analyzed it according to the WHO diagnosis (T-LGLL vs. CLPD-NK) or *STAT3* mutational status, in agreement with other published data [13,15]. Only a study reported by Barilà et al. found that patients with a STAT3 mutation had a lower OS [18].

As noted by other authors, neutropenia was present in approximately half of the patients and anemia in 25% of them [13,15,17,18]. Organomegaly was not a relevant feature of the disease. Remarkably, 37% of patients had a concomitant hematological disease. MGUS was the most frequent hematological disorder, found in about half of the cases, followed by a B-cell lymphoproliferative disorder. Other concomitant diseases were autoimmune disorders (21%), solid tumors (19%) and prior allogeneic stem cell transplantation (10%). Our data confirm those of Sanikommu et al. and Barilà et al., who documented the coexistence of MGUS, RA and neoplasms in similar proportions [13,18].

There was heterogeneity in the immunophenotype in the group of T-LGLL, with the most common profile being CD8^+^CD4^−^CD57^+^ and CD4^+^CD8^−^ or CD8^+/−^ less common, as previously described [19,20,21]. It is becoming apparent that these two immunological subgroups are indeed clinically and biologically distinct. Expansions of CD4^+^ LGL rarely manifest neutropenia or autoimmune diseases, but frequently co-occurred with solid tumors and often displayed *STAT5B* but not *STAT3* mutations. In contrast, neutropenia and *STAT3* mutations were almost exclusively found in CD8^+^ cases [19,21]. In our series, patients with CD8^+^CD57^+^ had a higher incidence of neutropenia, thrombocytopenia and lymphocytosis than the other immunological subtypes, but the differences were not statistically significant. Furthermore, all patients who needed treatment belonged to the CD8^+^CD57^+^ group (*p* = 0.032). Remarkably, patients included in the CD4^+^ group had a higher association with solid tumors (*p* = 0.037) compared to the CD8^+^CD4^−^ group. Nevertheless, these data should be taken carefully, since the numbers are relatively low and multiple comparisons were carried out.

Information on CLPD-NK is limited. Poullot et al [22] compared the clinical manifestations of 70 cases of CLPD-NK with those of T-LGLL and found that CLPD-NK patients were less symptomatic, rarely had RA and had less severe neutropenia and fewer infections, while they were more often associated with autoimmune cytopenia and solid tumors. Even though our patients with CLPD-NK had more frequently autoimmune diseases and solid tumors, the differences with T-LGLL were not statistically significant. Some authors have proposed that both entities could correspond to the same disease [23]. However, although it is obvious that there is some overlap in the clinical features between both entities, there are also subtle differences and the cell origin of the LGLL is clearly different. Therefore, with the information available so far, such as other authors, we cannot affirm that they are the same entity. At present, it has not been shown that both entities require a different clinical management. Therefore, with the information available so far, like other authors, we cannot affirm that they are the same entity.

Mutations in the *STAT3* gene were found in 17% of our patients. Y640F and D661Y were the most frequent mutations, in 45% and 35% of cases detected, respectively. Previous studies reported a higher prevalence of this mutation in T-LGLL; for example, Sanikommu et al. reported 36% and Rajala et al. reported 43% [13,17]. These differences could be due to the fact that *STAT3* mutations in these studies were analyzed by next-generation sequencing. This technique has higher sensitivity than Sanger sequencing, the technique used in our study [13,17]. In this context, Barilà et al [18]. and Teramo et al., [19]. who used Sanger sequencing, reported a prevalence of 28% and 37%, respectively. The differences in the frequency of *STAT3* mutation between these authors and our data can be explained by the fact that The differences in frequency of the STAT3 mutation reported between these authors and us could be explained by the fact that Barilà et al. analyzed exons 19–21 for *STAT3* [18]. and Teramo et al. also used ARMS-PCR (amplification refractory mutation system) for Y640F and D661Y, undetectable by Sanger sequencing [19]. Indeed, the frequency in our study may also be lower because we did not divide the mutational status of *STAT3* into different populations of expanded T/NK-LGL. Interestingly, and consistent with other published series, [13,18]. when we compared groups of patients according to the mutational status of *STAT3*, we observed a higher frequency of neutropenia, anemia and treatment requirement in those with mutated *STAT3*.

Few prospective trials have been carried out on LGLL and there is a lack of randomized trials to see which immunosuppressive drug has greater efficacy [16]. Algorithm treatment is not clearly defined in these entities, although indications for therapy are well known. In our series, only 14% of patients received treatment. This is in contrast to other reported series, [13,15]. where a higher proportion of patients was treated. A possible explanation is that, in those series, the prevalence of RA was higher than in ours and this is a treatment criterion to initiate immunosuppressive therapy [14]. However, our treatment response rate was similar to that reported in other studies in which methotrexate was the main pharmacological tool used, with an overall response rate of 50% [16,18]. There have been several reports that documented a correlation between the presence of *STAT3* mutations and neutropenia, treatment requirement and response to methotrexate in patients with the mutation affecting Y640F [16,19]. Our data confirm a higher need of treatment in the mutated cases, but we did not find an association between the response to methotrexate and the type of mutation.

In conclusion, our study extends the understanding of these two rare diseases and shows clinical similarities but also subtle differences between them. Our data further support the findings of others in terms of the clinical behavior of the disease and the biological significance of *STAT3* mutations. For the first time, we have shown that the survival of patients with these two diseases does not differ from that of a matched general Spanish population.

## Figures and Tables

**Table 1:** Main clinicobiological features of the 131 patients included in the study.

Variable	All Patients	T-LGLL	CLPD-NK
	*n* = 131	*n* = 97 (74%)	*n* = 34 (26%)
Sex (Male/Female), *n*	61/70	46/51	15/19
Age > 60 years, *n* (%)	90 (69)	67 (69)	23 (68)
B symptoms, *n* (%)	3 (2)	2 (1)	1 (3)
Splenomegaly, *n* (%)	16 (12)	12 (12)	4 (12)
Hepatomegaly, *n* (%)	3 (2)	2 (2)	1 (3)
Lymphadenopathy, *n* (%)	3 (2)	1 (1)	0
Lymphocytes *×* 10^9^/L, median (range)	3.9 (0.6–20.2)	4.37 (0.7–16.4)	3.8 (0.6–20.2)
LGL *×* 10^9^/L, median (range)	1.46 (0.12–18.98)	1.5 (0.12–13.9)	1.31 (0.14–18.9)
Hematologic manifestations, *n* (%)			
Neutrophils < 2.5 *×* 10^9^/L	70 (53)	54 (56)	16 (47)
Neutrophils < 1 *×* 10^9^/L	20 (15)	15 (15)	5 (15)
Neutrophils < 0.5 *×* 10^9^/L	4 (3)	4 (4)	0
Hemoglobin < 120 g/L	33 (25)	23 (24)	10 (29)
Hemoglobin < 80 g/L	1 (1)	1 (1)	0
Platelets < 130 *×* 10^9^/L *	22 (17)	17 (18)	5 (15)
Platelets < 50 *×* 10^9^/L	4 (3)	4 (4)	0
Raised LDH, *n* (%)	21/128 (16)	13/95 (14)	8/33 (24)
Raised β2-microglobulin, *n* (%)	52/79 (66)	39/61 (64)	13/18 (72)
Serum IgG > 15 g/L, *n* (%)	30/101 (30)	21/76 (28)	9/25 (36)
Autoimmune conditions, *n* (%)	22 (17)	15 (15)	7 (21)
Hematological disorders, *n* (%)	48 (37)	36 (37)	12 (35)
Solid tumors, *n* (%)	20 (15)	13 (13)	7 (21)
IgG+ for CMV, *n* (%)	49 (37)	37 (38)	12 (35)
Infections, *n* (%)	5 (4)	5 (100)	0
Respiratory tract infection		3 (60)	
Urinary tract infection		1 (20)	
*C. difficile* colitis		1(20)	
Patients needing treatment, *n* (%)	18 (14)	13 (13)	5 (15)

* Normal value of platelets in our laboratory: 130–400 × 10^9^/L; T-LGLL: T-cell large granular lymphocytic leukemia; CLPD-NK: chronic lymphoproliferative disorder of NK cells; LGL: large granular lymphocytes; LDH: lactate dehydrogenase; CMV: cytomegalovirus.

**Table 2:** In this case, 105 patients (80%) had diseases associated with T-LGL and CLPD-NK. Here, 105 patients had diseases associated with T-LGL and CLPD-NK.

Associated Disease	*n* = 105	T-LGLL	CLPD-NK
		*n* = 76	*n* = 29
Hematological disorders, *n* (%)	48 (46)	36	12
Monoclonal gammopathy	20 (42)	16	4
B-cell lymphoproliferative disorders *	19 (40)	14	5
MDS/AML	7 (14)	5	2
Acquired aplastic anemia	2 (4)	1	1
Autoimmune conditions, *n* (%)	22 (21)	15	7
Rheumatoid arthritis	5 (23)	2	3
Autoimmune haemolytic anemia	4 (18)	3	1
Immune thrombocytopenia	5 (23)	5	0
Others **	8 (36)	5	3
Solid tumors, *n* (%)	20 (19)	13	7
Urologic tumors	8 (40)	6	2
Gastrointestinal tumor	5 (25)	2	3
Breast cancer	2 (10)	1	1
Others ***	5 (25)	4	1
Transplantation			
Allogeneic stem cell transplantation, *n* (%)	11 (10)	9	2
Solid organ transplant, *n* (%)	3 (2)	2	1
Pulmonary hypertension, *n* (%)	1 (1)	1	0

T-LGLL: T-cell large granular lymphocytic leukemia; CLPD-NK: chronic lymphoproliferative disorder of NK cells; MDS: myelodysplastic syndrome; AML: acute myeloid leukemia; * 6 chronic lymphocytic leukemia/monoclonal B-cell lymphocytosis; 4 diffuse large B-cell lymphoma; 3 follicular lymphoma; 2 splenic marginal zone B-cell; 2 Waldenström macroglobulinemia; 1 Hairy cell leukemia;1 Hodgkin lymphoma; ** Others: autoimmune hepatitis, pemphigus, Sjogren syndrome, ulcerative colitis, autoimmune thyroiditis; *** Other neoplasias: ovarian cancer, tongue cancer and skin non-melanoma cancer.

**Table 3:** Clinicobiological characteristics according to phenotype of T-LGLL.

Variable	CD8^+^CD57^+^	CD4^+^	*p*-Value
	*n* = 72	*n* = 22	
Sex (Female/Male)	35/37	13/9	NS
Age > 60 years, *n* (%)	46 (64)	18 (81)	NS
B symptoms, *n* (%)	2 (3)	0	NS
Splenomegaly, *n* (%)	10 (14)	2 (9)	NS
Hepatomegaly, *n* (%)	2 (3)	0	NS
Lymphadenopathy, *n* (%)	0	1 (5)	NS
Lymphocytosis (>4.5 × 10^9^/L), *n* (%)	36 (50)	8 (36)	NS
LGL (>2 × 10^9^/L), *n* (%)	21 (29)	5 (23)	NS
Hematologic manifestations, *n* (%)			
Hemoglobin < 12 g/L	17 (24)	5 (23)	NS
Neutrophils < 2.5 × 10^9^/L	42 (58)	9 (41)	NS
Platelets < 130 × 10^9^/L	16 (22)	1 (5)	0.059
Raised LDH, *n* (%)	10 (14)	2 (9)	NS
Raised β2-microglobulin, *n* (%)	29 (40)	9 (41)	NS
Autoimmune conditions, *n* (%)	11 (15)	3 (14)	NS
Hematological disorders, *n* (%)	26 (36)	9 (41)	NS
Solid tumors, *n* (%)	7 (10)	6 (27)	0.037
*STAT3* mutated, *n* (%)	13/67 (19)	1/21 (5)	NS
Patients needing treatment, *n* (%)	13 (18)	0	0.032

NS: not significant; LGL: large granular lymphocytes; LDH: lactate dehydrogenase.

**Table 4:** Clinicobiological characteristics according to the mutational status of *STAT3*.

Variable	STAT3*^wt^*	STAT3*^mut^*	*p*-Value
	*n* = 99	*n* = 20	
Sex (Female/Male)	54/45	11/9	NS
Age > 60 years, *n* (%)	65 (66)	16 (80)	NS
Subtype (WHO 2017), *n* (%)			
T-LGLL	76 (77)	15 (75)	
CLPD-NK	23 (23)	5 (25)	NS
B symptoms, *n* (%)	3 (3)	0	NS
Splenomegaly, *n* (%)	12 (12)	4 (20)	NS
Hepatomegaly, *n* (%)	3 (3)	0	NS
Lymphadenopathy, *n* (%)	1 (1)	0	NS
Lymphocytosis (>4.5 × 10^9^/L), *n* (%)	38 (38)	11 (55)	NS
LGL (>2 × 10^9^/L), *n* (%)	19/68 (28)	7/15 (47)	0.039
Hematologic manifestations, *n* (%)			
Hemoglobin < 12 g/L	23 (23)	9 (45)	0.046
Neutrophils < 2.5 × 10^9^/L	49 (49)	17 (85)	0.005
Platelets < 130 × 10^9^/L	17 (17)	4 (20)	NS
Raised LDH, *n* (%)	10 (10)	8 (40)	0.003
Raised β2-microglobulin, *n* (%)	39 (39)	11 (55)	NS
Autoimmune conditions, *n* (%)	16 (16)	3 (15)	NS
Hematological disorders, *n* (%)	39 (39)	6 (30)	NS
Solid tumors, *n* (%)	15 (15)	4 (20)	NS
Patients needing treatment, *n* (%)	12 (12)	6 (30)	0.042

NS: not significant; LGL: large granular lymphocytes; T-LGLL: T-cell large granular lymphocytic leukemia; CLPD-NK: chronic lymphoproliferative disorder of NK cells; LDH: lactate dehydrogenase.

## Data Availability

The data presented in this study are available in the article.

## Supplementary Materials

The following are available online at https://www.mdpi.com/article/10.3390/cancers13153900/s1, Table S1: List of antibodies employed in the diagnosis of T-cell large granular lymphocytic leukemia and chronic lymphoproliferative disorder of NK cells.

## References

[1] Campo E., Harris N.L., Jaffe E.S., Pileri S.A., Stein H., Thiele J., Vardiman J.W., Swerdlow S.H. (2017). WHO Classification of Tumours of Haematopoietic and Lymphoid Tissues.

[2] Shah M.V., Hook C.C., Call T.G., Go R.S. (2016). A population-based study of large granular lymphocyte leukemia. Blood Cancer J..

[3] Dinmohamed A.G., Brink M., Visser O., Jongen-Lavrencic M. (2016). Population-based analyses among 184 patients diagnosed with large granular lymphocyte leukemia in the Netherlands between 2001 and 2013. Leukemia.

[4] Matutes E. (2017). Large granular lymphocytic leukemia. Current diagnostic and therapeutic approaches and novel treatment options. Expert Rev. Hematol..

[5] Moignet A., Lamy T. (2018). Latest Advances in the Diagnosis and Treatment of Large Granular Lymphocytic Leukemia. Am. Soc. Clin. Oncol. Educ. B.

[6] Fasan A., Kern W., Haferlach C., Haferlach T., Schnittger S. (2012). STAT3 Mutations in Large Granular Lymphocytic Leukemia. Blood.

[7] Jerez A., Clemente M.J., Makishima H., Koskela H., LeBlanc F., Peng Ng K., Olson T., Przychodzen B., Afable M., Gomez-Segui I. (2012). STAT3 mutations unify the pathogenesis of chronic lymphoproliferative disorders of NK cells and T-cell large granular lymphocyte leukemia. Blood.

[8] Rajala H.L., Eldfors S., Kuusanmäki H., Van Adrichem A.J., Olson T., Lagström S., Andersson E.I., Jerez A., Clemente M.J., Yan Y. (2013). Discovery of somatic STAT5b mutations in large granular lymphocytic leukemia. Blood.

[9] Rajala H.L.M., Mustjoki S. (2013). STAT5b in LGL leukemia-a novel therapeutic target?. Oncotarget.

[10] Dippel E., Assaf C., Hummel M., Schrag H.J., Stein H., Goerdt S., Orfanos C.E. (1999). Clonal T-cell receptor γ-chain gene rearrangement by PCR-based genescan analysis in advanced cutaneous T-cell lymphoma: A critical evaluation. J. Pathol..

[11] Van Dongen J.J.M., Langerak A.W., Brüggemann M., Evans P.A.S., Hummel M., Lavender F.L., Delabesse E., Davi F., Schuuring E., García-Sanz R. (2003). Design and standardization of PCR primers and protocols for detection of clonal immunoglobulin and T-cell receptor gene recombinations in suspect lymphoproliferations: Report of the BIOMED-2 concerted action BMH4-CT98-3936. Leukemia.

[12] Raffeld M. (2014). Frequent STAT5B mutations in γδ hepatosplenic T-cell lymphomas. Leukemia.

[13] Sanikommu S.R., Clemente M.J., Chomczynski P., Afable M.G., Jerez A., Thota S., Patel B., Hirsch C., Nazha A., Desamito J. (2018). Clinical features and treatment outcomes in large granular lymphocytic leukemia (LGLL). Leuk. Lymphoma.

[14] Lamy T., Moignet A., Loughran T.P. (2017). LGL leukemia: From pathogenesis to treatment. Blood.

[15] Bareau B., Rey J., Hamidou M., Donadieu J., Morcet J., Reman O., Schleinitz N., Tournilhac O., Roussel M., Fest T. (2010). Analysis of a French cohort of patients with large granular lymphocyte leukemia: A report on 229 cases. Haematologica.

[16] Loughran T.P., Zickl L., Olson T.L., Wang V., Zhang D., Rajala H.L., Hasanali Z., Bennett J.M., Lazarus H.M., Litzow M.R. (2015). Immunosuppressive therapy of LGL leukemia: Prospective multicenter phase II study by the Eastern Cooperative Oncology Group (E5998). Leukemia.

[17] Rajala H.L., Olson T., Clemente M.J., Lagström S., Ellonen P., Lundan T., Hamm D.E., Zaman S.A.U., Marti J.M.L., Andersson E.I. (2015). The analysis of clonal diversity and therapy responses using STAT3 mutations as a molecular marker in large granular lymphocytic leukemia. Haematologica.

[18] Barilà G., Teramo A., Calabretto G., Vicenzetto C., Gasparini V.R., Pavan L., Leoncin M., Vedovato S., Frigo A.C., Facco M. (2020). Stat3 mutations impact on overall survival in large granular lymphocyte leukemia: A single-center experience of 205 patients. Leukemia.

[19] Teramo A., Barilà G., Calabretto G., Ercolin C., Lamy T., Moignet A., Roussel M., Pastoret C., Leoncin M., Gattazzo C. (2017). STAT3 mutation impacts biological and clinical features of T-LGL leukemia. Oncotarget.

[20] Muñoz-García N., Jara-Acevedo M., Caldas C., Bárcena P., López A., Puig N., Alcoceba M., Fernández P., Villamor N., Flores-Montero J.A. (2020). STAT3 and STAT5B mutations in T/NK-cell chronic lymphoproliferative disorders of large granular lymphocytes (LGL): Association with disease features. Cancers.

[21] Andersson E.I., Tanahashi T., Sekiguchi N., Gasparini V.R., Bortoluzzi S., Kawakami T., Matsuda K., Mitsui T., Eldfors S., Bortoluzzi S. (2016). High incidence of activating STAT5B mutations in CD4-positive T-cell large granular lymphocyte leukemia. Blood.

[22] Poullot E., Zambello R., Leblanc F., Bareau B., De March E., Roussel M., Boulland M.L., Houot R., Renault A., Fest T. (2014). Chronic natural killer lymphoproliferative disorders: Characteristics of an international cohort of 70 patients. Ann. Oncol..

[23] Zambello R., Teramo A., Gattazzo C., Semenzato G. (2014). Are T-LGL Leukemia and NK-Chronic Lymphoproliferative Disorder really two distinct diseases?. Transl. Med. UniSa Action.

