# Supplementary material for: Clinicobiological Characteristics and Outcomes of Patients with T-Cell Large Granular Lymphocytic Leukemia and Chronic Lymphoproliferative Disorder of Natural Killer Cells from a Single Institution
Source: Cancers (Basel). 2021 Aug 2;13(15):3900. doi: 10.3390/cancers13153900 (PMC8345581; doi:10.3390/cancers13153900)
Supplement: Supplementary file 1 [file cancers-13-03900-s001.zip › cancers-1303462-supplementary.pdf]

cancers MDPI

Supplementary material: Clinicobiological Characteristics and Outcomes of Patients with T-Cell Large Granular Lymphocytic Leukemia and Chronic Lymphoproliferative Disorder of Natural Killer Cells from a Single Institution

Andrea Rivero, Pablo Mozas, Laura Jiménez, Mónica López-Guerra, Dolors Colomer, Alex Bataller, Juan Correa, Alfredo Rivas-Delgado, Gabriela Bastidas, Tycho Baumann, Alejandra Martínez-Trillos, Julio Delgado, Eva Giné, Elías Campo, Armando López-Guillermo, Neus Villamor, Laura Magnano and Estella Matutes

Table S1. List of antibodies employed in the diagnosis of T-cell large granular lymphocytic leukemia and chronic lymphoproliferative disorder of NK cells.

Cell Type	Antibody
B lymphocytes	CD19
T lymphocytes	CD3, CD4, CD8, CD2, CD5, CD7, TCRαβ, TCRγδ, CD57
NK Cells	CD56, CD16
